# Establishing a Core Domain Set for early-phase clinical trials of electrical stimulation interventions for tinnitus in adults: protocol for an online Delphi study

**DOI:** 10.1186/s13063-022-07020-2

**Published:** 2022-12-21

**Authors:** Bas Labree, Derek J. Hoare, Kathryn Fackrell, Deborah A. Hall, Lauren E. Gascoyne, Magdalena Sereda

**Affiliations:** 1 NIHR Nottingham Biomedical Research Centre, Nottingham, UK; 2 Hearing Sciences, Mental Health and Clinical Neuroscience, University of Nottingham, Nottingham, UK; 3 Wessex Institute, University of Southampton, Southampton, UK; 4 Department of Psychology, School of Social Sciences, Heriot-Watt University, Putrajaya, Malaysia; 5 Sir Peter Mansfield Imaging Centre, School of Physics and Astronomy, University of Nottingham, Nottingham, UK

**Keywords:** Tinnitus, Neuromodulation, Core Domain Set

## Abstract

**Background:**

Tinnitus is the awareness of a sound in the ear or head in the absence of an external source. It affects around 10–15% of people and current treatment options are limited. Experimental treatments include various forms of electrical stimulation of the brain. Currently, there is no consensus on the outcomes that should be measured when investigating the efficacy of this type of intervention for tinnitus. This study seeks to address this by establishing a Core Domain Set: a common standard of what specific tinnitus-related complaints are critical and important to assess in all clinical trials of electrical stimulation-based interventions for tinnitus.

**Methods:**

A two-round online survey will be conducted, followed by a stakeholder consensus meeting to identify a Core Domain Set. Participants will belong to one of two stakeholder groups: healthcare users with lived experience of tinnitus, and professionals with relevant clinical, commercial, or research experience.

**Discussion:**

This study will establish a Core Domain Set for the evaluation of electrical stimulation-based interventions for tinnitus via an e-Delphi study. The resulting Core Domain Set will act as a minimum standard for reporting in future clinical trials of electrical stimulation interventions for tinnitus. Standardisation will facilitate comparability of research findings.

## Background

Tinnitus is the sensation of noise, ringing, buzzing or hissing sound perceived in the ears or head [1]. In most cases, tinnitus is only perceived subjectively. Approximately 65 million people in Europe [2] and more than 50 million people in the United States experience tinnitus. Tinnitus can be chronic and disabling and is associated with a diverse range of complaints, including perceived loudness, sleep problems, difficulties in listening and concentration, effects on psychological well-being, daily life and on general health [3–6]. Tinnitus may also negatively affect the quality of life and has a societal impact in terms of social withdrawal and impaired work performance [7, 8]. Each of these complaints could be defined as a distinct domain of tinnitus. Currently, there are no objective assessment tools to measure the impact or severity of tinnitus. Assessment, diagnosis, and evaluation are entirely reliant on self-report. Many multi-item tinnitus questionnaires have been published over the past few decades and are often used as outcome measures to evaluate the effectiveness of interventions [9]. For further details on the validity of commonly used self-report measures of tinnitus (Tinnitus Questionnaire, Tinnitus Handicap Inventory, Tinnitus Functional Index and Visual Analogue Scales), see [10–12]. In the context of clinical trials, an outcome is a measurement or observation used to assess the effectiveness or risk (such as side effects) of an intervention. For instance, outcomes collected in trials of tinnitus include tinnitus intrusiveness, tinnitus loudness, annoyance, intensity and distress [13]. Therefore, it is perhaps unsurprising that across different trials of tinnitus interventions the domain outcomes and measures reported varies widely.

A set of outcome domains and instruments that have been agreed upon for a health condition is called a Core Outcome Set. The purpose of a Core Outcome Set is to define a minimum set of outcomes to be measured in every trial of a particular type of intervention in a specific area of health — in this case, electrical stimulation-based interventions for tinnitus. This does not necessitate that outcomes in a particular trial should be restricted to only those in the Core Outcome Set. Rather, there is an expectation that the Core Outcome Set will always be collected and reported, but additional outcomes can be measured. Defining what domains are important to measure will create the Core Domain Set which is the crucial first step in this process. It is important to identify outcome domains that are appropriate for the intervention strategy to have confidence that if a trial showed no effect, it did so because the intervention was not effective, not because the outcomes measured were inappropriate for that particular intervention. A Core Domain Set developed from the perspectives of healthcare users and professionals would address this. Perspectives of healthcare users with the lived experience of the condition are important for understanding what matters to them. Instances have arisen where patients have identified outcomes as important that were previously overlooked [14] or thought to be of little importance [15, 16]. Not every Core Domain Set has been developed with healthcare users’ input. A recent systematic review of patient participation in developing Core Domain Sets found variability in study methods with no clear evidence on how to best promote patient recruitment [17]. However, studies that involve patients in the study design are at least reasonably well placed to consider enablers and barriers to public participation at the study design phase.

Currently, no treatment exists that eliminates tinnitus but many interventions are being trialled. A recent systematic review, examining 228 published clinical trials, suggested that there are eight broad classes of intervention strategies currently being tested worldwide [18]. Each of these intervention strategies is aimed at alleviating different outcome domains of tinnitus. For example, psychological interventions are intended to reduce emotional distress. The previously mentioned systematic review identified 35 primary domain outcomes spanning seven broad categories (tinnitus percept, impact of tinnitus, co-occurring complaints, quality of life, body structures and function, treatment-related outcomes and unclear or not specified). The most commonly reported domains, out of 228 articles, were tinnitus loudness (14% as a primary outcome and 7% as secondary outcome) and tinnitus distress (7% as a primary outcome and 3% as secondary outcome). The method of assessment for these outcome domains varied between studies, even when the same treatment outcome domain was being evaluated. For example, loudness was measured using either a numerical rating scale (8%), loudness matching (4%), minimum masking level (1%) and loudness discomfort level (1%). This lack of a standardised assessment can severely hinder identification and interpretation of the relative merits of the various treatments that are currently on offer or interventions under investigation, and the most appropriate approaches for individual patients [19].

The Core Outcome Measures in Tinnitus: International Delphi (COMiT’ID) study identified separate Core Domain Sets for sound-, psychology-, and pharmacology-based interventions for tinnitus [13]. The most commonly reported intervention strategies identified through the systematic review [18] were pharmacological (*n* = 66), electrophysiological (*n* = 59), sound therapy (*n* = 56) and psychological (*n* = 47). At the time of the COMiT’ID study [13] electrophysiology interventions were excluded because available evidence was limited and dominated by a particular research group, whilst the other three encompassed broad international efforts in clinical research for tinnitus [18]. A few years on, the professional community has increased its efforts to develop electrophysiological treatment options and more clinical trials are being conducted. It is now appropriate to develop a Core Domain Set for this type of intervention, using comparable methodology. Therefore, this study seeks to build on previous work [13] to develop a Core Domain Set for electrical stimulation-based interventions for tinnitus.

### Scope

For the purposes of the Core Outcome Measures in Tinnitus: Electrical Stimulation (COMiT-ES) study, electrical stimulation-based interventions for tinnitus are defined as treatment that aims to improve tinnitus or its symptoms by electrical stimulation of the brain or other parts of the nervous system. This means that techniques that do not have a proposed mechanism that operates via the brain, or that do not have improvements in tinnitus symptoms as their primary aim, are excluded. Furthermore, this Core Domain Set will not cover devices such as cochlear implants or Transcranial Magnetic Stimulation (TMS) that convert sound or magnetic energies into electrical pulses. Non-invasive brain stimulation methods such as transcranial direct current stimulation (tDCS), transcranial alternating current stimulation (tACS) and transcranial random noise stimulation (tRNS) are included, and invasive methods such as vagus nerve stimulation (VNS) will be covered. This study will result in an electrical stimulation-specific Core Domain Set that should be measured and reported in all clinical trials evaluating electrical stimulation interventions for tinnitus.

The aim of this study is to determine a core outcome domain set for electrical stimulation-based interventions for tinnitus. The objectives of this study are therefore to compare and integrate perspectives on outcome domains for electrical stimulation-based interventions for tinnitus through (1) a Delphi survey, and (2) a consensus meeting with stakeholders. Key stakeholders are professionals working in the field of tinnitus (clinical practitioners, clinical researchers, commercial representatives and funders), and members of the public with lived experience of tinnitus.

## Methods

This study is sponsored by the University of Nottingham and co-ordinated and managed by the National Institute of Health Research (NIHR) Nottingham Biomedical Research Centre (BRC). This protocol was approved by Yorkshire and the Humber – Sheffield Research Ethics Committee.

### Research Advisory Group

A Research Advisory Group has been appointed to guide the project and aid in decision-making. The group comprises experts in the fields of tinnitus and Core Outcome Set development (DAH, KF), a Patient and Public Involvement and Engagement Manager and the Study Management Team (BL, DJH, LEG, MS). The role of the Research Advisory Group is as follows:

(i) Supported the development of the study protocol by providing feedback.
(ii) Participate in online meetings to discuss progress on the Delphi study
(iii) Review study documentation, including information sheets for members of the public and professionals and intended advertisements
(iv) participate in the piloting of the survey

None of the members of the Research Advisory Group will be allowed to vote on domains in the final consensus meeting. Any conflicts of interest within the Study Management Team will be described in the final report, including a brief summary of how these were managed (Kirkham et al., 2016).

### Eligibility criteria for the online surveys

We will include representatives from two stakeholder groups with relevant experience and/or interest in electronic stimulations for management of chronic subjective tinnitus in adults. To be eligible for participation participants must be aged 18 or over and have a sufficient command of English to read, understand, and complete questionnaires independently. Specific inclusion criteria defined for each group are as follows:

Healthcare users with lived experience of tinnitus (healthcare user stakeholders) must have experience of living with tinnitus for 3 months or more and have received a form of electrical stimulation for tinnitus (such as tDCS, tACS, tRNS, or direct nerve stimulation) or would consider trying this type of treatment for their tinnitus.

Professional stakeholder group will include representatives from healthcare, clinical research, commercial sector, and research funders. A targeted recruitment strategy will be used to ensure the inclusion of an equal number of researchers, clinicians, and commercial representatives. These professionals have been identified as representing the main professional categories in tinnitus research and clinical trials that would have representative homogenous samples. Therefore, to be eligible professionals stakeholders must meet one of the following criteria:

- Have a clinical qualification, be currently employed by a public or private institution that provides a tinnitus service to patients, have experience of assessing, diagnosing or managing chronic subjective tinnitus, have a working knowledge and/or clinical experience of electrical stimulation for tinnitus
- Have an academic qualification, be currently employed by a research organisation, have current or “recent past” experience with studies that focus on questions of clinical efficacy of a tinnitus intervention in humans, with specific focus on interventions involving electrical stimulation with “recent past” being defined as having been a co-author on a relevant peer-reviewed journal publication in the past three years
- Be currently employed by a company that develops, manufactures or sells products that involve electrical stimulation that may be trialled for effectiveness in alleviating tinnitus
- Be currently employed by an organisation that has funded tinnitus research projects addressing electrical stimulation-based interventions in the last three years

Journal editors will not be included as a separate stakeholder group because it would not be possible to meet the minimum sample size requirement due to the small population size. However, given that in some cases professional stakeholders within existing groups will have a secondary occupation of journal editor, this profession is still likely to be represented. The secondary role of journal editor will therefore be recorded, allowing for this sub group of participant data to be examined for any notable differences during the analysis phase.

During the introduction page of the online survey, both professionals and healthcare users will be asked to indicate informed consent and self-certify as being an ‘expert’ in electrical stimulation-based interventions for tinnitus based on our definition.

### Panel size and justification

There is no agreed method to statistically calculate a sample size for Delphi surveys or for consensus meetings and no criteria against which a sample size choice can be judged (e.g. Powell, 2003; Akins et al., 2005). Some individual studies indicate that stakeholder groups of around 20 can provide results that are representative of the views of the wider stakeholder group (Akins et al., 2005). However, one of the key deciding factors is that the participant panel membership should adequately represent their corresponding stakeholder group. Another one is pragmatic. One practical factor that will influence our sample size is the aim of a roughly equal number of participants in each stakeholder group. The consensus meeting will include 20 participants, in line with COMiT’ID [18]. For the Delphi survey and consensus meeting, there will be an equal number of participants from each stakeholder group, or as close as practically possible, and professionals will be balanced across the clinical, academic and commercial sectors.

### Recruitment methods

We will use an online Delphi process to gain consensus of opinion, which requires broadly representative professional and healthcare user stakeholder groups. We will use non-probabilistic purposive sampling to recruit 60 professionals (minimum 30) and 60 healthcare users (minimum 30) with experience in tinnitus for the Delphi survey, both from the UK and overseas. If the target number of participants is not recruited within the first 2 months of survey launch, then a minimum number of 20 in each stakeholder group will be accepted. In this eventuality, recruitment will continue until at least 20 participants are recruited to each group. To ensure a representative sample of participants with a wide range of experience and perspectives, several recruitment strategies (clinical and non-clinical) will be considered for patient stakeholders via both clinical and non-clinical routes.

*Clinical routes*

1. Participants will be recruited from NHS audiology and ENT services at Nottingham University Hospitals NHS Trust. The initial approach will be from a member of the patient’s usual care team or the NIHR Clinical Research Network (CRN) audiologist, who will provide the participant with a study information pack. The study information pack will contain:

i. An invitation letter
ii. The participant information sheet (PIS), explaining all aspects pertaining to participation in the study
iii. A reply slip to register interest in participating in the study (name, date of birth, postal and email addresses, telephone number, preferred participation method [online/post]).
iv. A prepaid envelope for reply slip return. Reply slips can also be completed in clinic and handed back to the NIHR CRN audiologist.

2. Posters and leaflets about the study will be on display in clinical areas (NHS audiology and ENT) directing interested patients to speak with the NIHR CRN audiologist or to contact the research team via email.
3. Additional national Participant Identification Centres (PICs) will be identified via the NIHR CRN to ensure the recruitment target can be met in a timely manner.

*Non-clinical routes*

1. Invitation emails will be sent to those registered on the NIHR Nottingham Biomedical Research Centre (Hearing Theme) database of research volunteers.
2. Newsletter articles and announcements will be published by relevant patient and professional organisations (e.g. Action on Hearing Loss, Hearing Link, British Society of Audiology).
3. Posts will be made on social media channels (Twitter, e.g.: @hearingnihr, @JLAhearing, @HearSci @NottmBRC, @UoNHearSci, @NUHMedicine) and Facebook (NIHR Nottingham BRC, Hearing Theme).
4. A Participant Information Centre (PIC) at the University of Nottingham will facilitate recruitment of participants in the Professionals stakeholder group using existing professional networks.

Professional stakeholders will be recruited via existing professional networks and by contacting professionals who can be deduced to meet the eligibility criteria, for instance, those who have been co-authors on a relevant peer-reviewed journal publication in the past three years, in the case of clinical researchers.

Recruitment will take place online, including in partnership with both national and international organisations. We will aim to recruit both healthcare users and professionals internationally. International recruitment of professionals will take place via the authors’ existing professional networks as well as via emails to corresponding authors listed on relevant recent publications. International recruitment of healthcare users will take place primarily via tinnitus forums and social media.

An electronic (or postal) version of the study information pack will be sent to individuals expressing an interest in participating in the study via non-clinical routes. The NIHR CRN audiologist or the Chief Investigator (CI) or Study Coordinator will request that candidate participants confirm they meet the criteria for inclusion. To reduce attrition, reminder e-mails will be sent. Participants will only be invited to take part in Round 2 if they have completed Round 1. They will only be invited to the consensus meeting if they have completed both survey rounds by responding to all outcome domains (i.e. rating 1–9 or selecting “unable to rate”).

To recruit for the consensus meeting, participant information sheets for the survey will inform participants to register their interest in attending the consensus meeting with the research team. Participants will be informed that there are only a limited number of places available. Participants will be reminded after completing the first questionnaire to register for the meeting if they wish to attend. If the required number of participants have not registered following the first questionnaire they will be reminded to register after they completed the second questionnaire. Allocation of places at the consensus meeting will be based on participants who participated in both rounds of the Delphi survey and registered an interest first. The email address and phone number of the Chief Investigator will be available to participants for questions and support. Participants in the consensus meeting will not be screened based on their responses in the survey rounds. Equal participation across stakeholder groups will be encouraged, but if this is not possible, the consensus meeting will go ahead with unequal numbers.

### Preparatory work

The COMiT’ID study generated a long list of outcomes via a systematic review [18] and narrative synthesis [9]. The systematic review that yielded the original long list of outcomes prior to COMiT’ID included all trials of interventions of any type for tinnitus. Following this, the authors decided to run Delphi studies for their three intervention types of interest and did not use the electrical stimulation-specific outcomes resulting from the systematic review in that study. However, this study will include those previously excluded outcomes. The definitions in this list were subject to PPI input from the stakeholder groups in this study [20]. The COMiT’ID study long list of outcomes may be modified for this study. Initially, the long list will be inspected and reviewed by members of the Study Management Team (MS, DH and BL). Domains that have numerous different conceptualisations, or reflect complex composite complaints, may be combined based on the COMiT’D forum study results [21] or may be removed if deemed irrelevant to electrical stimulation interventions. Exclusions will be based on discussions of the Study Management Team and Advisory Group. Any possible duplications of outcomes will be condensed, producing a final list of domains. The names and definitions of the outcome domains, which were reviewed by PPI as part of the COMiT’ID study, will not be reviewed again. New materials, such as Participant Information Sheets were reviewed by members of the NIHR Nottingham BRC Hearing Theme PPI group. Members of the PPI group will be asked to navigate the survey software and read the contents of the survey ahead of the launch of Round 1 of the Delphi survey to test usability and face validity. Our PPI manager advised on the PPI strategy and facilitated contact with the PPI group ahead of submission to the ethics committee.

All outcome domains have plain language descriptions, generated for each outcome using an iterative process as part of the COMiT’ID study [20]. The purpose of the plain language descriptions is to ensure that all domains are correctly interpreted by all stakeholders, including public participants, therefore facilitating accurate and consistent understanding of domains across participant groups. To facilitate presentation of the final long list, outcome domains will be categorised into over-arching domains. The final long list of categorised outcome domains will be operationalised into questionnaire items (with the plain language description).

Reporting of the Delphi survey will fully describe how outcomes were added, removed or condensed, with reasons [22]. It will also list all outcome domains considered at the start of the consensus process [22].

### The Delphi survey

**Fig. 1:**
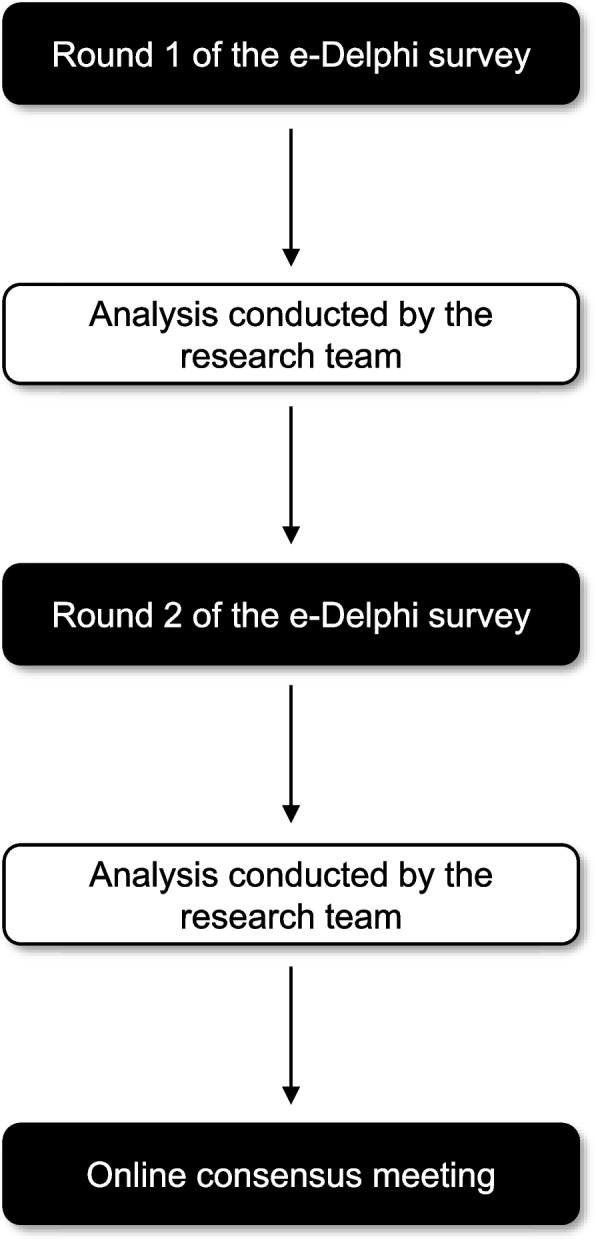
Overview of the study plan

The Delphi survey will comprise two sequential questionnaires or ‘rounds’ aiming to obtain a consensus of opinion from professional and healthcare user stakeholder groups. The Delphi survey will be managed using DelphiManager, a bespoke online e-management system maintained by the COMET Initiative [23, 24]. Each survey round will contain a questionnaire that includes the final long list of categorised outcome domains developed as described above (Fig. 1).

#### Round 1

For each questionnaire item, participants will be asked to think about the importance of a tinnitus outcome domain and indicate how important that domain is to measure when deciding whether an electrical stimulation-based tinnitus treatment is working. The outcome domains will appear in a randomised order so as to avoid order effects, while the outcomes within them will be listed in alphabetical order to avoid potential weighting [25]. Participants will be asked to score each outcome domain using the GRADE scale of 1–9, where 1 represents least important and 9 represents most important [26]. Selecting response options 1–3 will be taken to indicate that the domain is considered ‘not important’, while 7–9 will be taken to indicate that the domain is ‘critically important’ in deciding whether a tinnitus treatment is having its desired effect. Scores 4, 5 and 6 will be taken to indicate the outcome domain is considered “important but not critical”. If a participant feels that they did not understand a particular outcome, they will be able to select “unable to score”.

Participants will have the option to suggest additional outcomes domains for inclusion in Round 2. These additional outcomes will be reviewed and coded by two Study Management Team members to ensure they represent new outcomes. Where uncertainty exists, the rest of the team will be consulted. Definitions will be generated for the new outcomes and reviewed by the Study Management Team. Members of the Research Advisory Group may also be consulted if appropriate. Reporting of the Delphi survey will describe any new outcomes introduced into the consensus process at the end of Round 1, with reasons [22]. Following each outcome and at the end of the questionnaire, each participant will be offered an open-text box to add any comments about particular outcome domains. This will be optional. The distribution of the scores for each outcome domain will be calculated for each stakeholder group within the Delphi survey.

#### Round 2

The purpose of Round 2 is to enable participants to reflect on their scores considering the viewpoint of their own stakeholder group and the other stakeholder group in the Delphi survey. In the second round, participants will be presented with the same list of outcome domains as in Round 1, and any new outcomes identified by participants in Round 1. Participants will see the same list of outcome domains with their own previous score, the anonymised distribution of scores across their stakeholder group and the anonymised distribution of scores from the other stakeholder groups from Round 1. Results will be presented graphically as well as numerically to improve visual appeal. Participants will be asked to re-score the same list of outcome domains, considering this new information. To help give meaning to the GRADE scale, participants will be reminded that any outcome domain will only be considered for inclusion in the Core Domain Set if 70% of participants in each group select points 7–9 on the scale, and less than 15% select points 1–3.

### The online consensus meeting

Professionals and healthcare users who have completed both rounds of the Delphi survey and registered an interest in participating in the consensus meeting will be allocated places based on a first come, first served basis. In the case of the professional stakeholders, efforts will be made to ensure clinicians, researchers and commercial representatives are represented. In advance of the meeting, participants will be asked to vote on whether or not the scope of the consensus meeting should be limited to the outcomes achieving consensus in Round 2 of the Delphi survey.

To minimise screen time during the web-based consensus meeting, participants will be supplied with an overview of the aims of the day and a guidance document outlining the day’s activities. Participants will be asked to complete an online survey identifying their personal top three outcomes from those achieving consensus from the e-Delphi. The purpose of this is to steer both the participants’ thoughts and the conversation in the meeting towards what outcomes are critical. Experienced independent moderators will be recruited to facilitate the consensus meeting discussion to agree a final Core Domain Set. The consensus meeting will be audio-recorded and transcribed to facilitate reporting. These will be classed as source data and will be retained in the study archives, using Unique Identifier Codes for each speaker. Reporting of the Delphi survey will list the outcomes in the final Core Domain Set [22]. The consensus meeting will comprise a plenary stage as well as breakout discussions in which the participants will be allocated to groups along stakeholder lines. The consensus meeting will include anonymised voting on each outcome as either “In” or “Out” which will create histograms and descriptive statistics ‘live’, to be displayed in the meeting. In advance of the meeting, participants will be given materials summarising the anonymised Round 2 results, separately for each stakeholder group. If after two consecutive votes, the consensus criterion is not met for any outcome, they will be dropped in favour of a simple majority criterion, that is, outcomes will be included if more than 50% of participants vote for its inclusion. Because the time for discussion will be limited, there will be no discussion about outcomes that met the criteria for exclusion. In line with the Core Domain Sets resulting from the COMiT’ID study [13], the maximum number of outcomes in the Outcome Domain Set will be seven (adverse effects and up to six further outcomes). Therefore, while the Delphi survey will determine which outcomes are brought to the consensus meeting, if there are more than seven outcomes then the consensus meeting participants will be asked to reduce the number of outcomes. All Delphi survey participants will be invited to agree or disagree with the decisions made at the consensus meeting and these results will be disseminated along with the Core Outcome Domain Set.

### Consensus criteria Delphi survey rounds

Consensus recommendations will be made according to the following definition [27, 28]:

- Include domain in Core Domain Set: 70% or more of the participants in each stakeholder groups score 7–9, and fewer than 15% score 1–3.
- Exclude outcome domains in Core Domain Set: 50% or fewer participants in each stakeholder group score 7–9

### Consensus criteria consensus meeting

Consensus from the meeting is defined as 70% or more of the participants agreeing on including one or more outcome domains in the Core Domain Set.

### Data analysis

Reporting of the Delphi survey will fully describe the relevant characteristics of the participants involved at each stage of the Core Domain Set development [22]. Examples of relevant characteristics are gender, ethnic background, socioeconomic status, country and region (e.g. UK (Wales), America (Iowa), France (Paris)), since these factors affect how representative the consensus might be of the target population. There are no special features for the proposed analysis. Descriptive statistics will be computed for scores on each domain (response distributions (%) of participants selecting each of the 1–9 Likert response options).

A designated member of the Study Management Team will analyse numerical data collected in the Delphi survey, using the UoN license for SPSS and Microsoft excel. The data from each round will be subjected to descriptive statistics, such as the distribution of the relevant participant characteristics, distribution of rating scores across each stakeholder group (including the “unable to score” option), and attrition rate from round to round. A separate analysis will assess the shifts in scores across rounds as a consequence of considering the anonymised feedback from other participants. Round 2 score distributions for each outcome domain will be considered at the final consensus meeting using a nominal group technique to evaluate individual perspectives

### Dissemination

Data from the final analysis of the Delphi, and consensus meeting will be presented at relevant national and international conferences, including the British Tinnitus Association and British Society of Audiology meetings. We intend to publish the results of this study in a peer-reviewed journal. This research will be further disseminated to members of the public and clinicians through specialist magazine articles and support groups. In addition, uptake of the Core Domain Set will be actively promoted through deposition in any relevant outcome depositories and informal dissemination within existing professional networks, including via social media channels. Participants will not be identified in any publications.

## Discussion

The purpose of this Delphi study is to establish a Core Domain Set-a list of outcomes that should inform the choice of measurements used when trialling electrical stimulation-based interventions for tinnitus. In order to ensure this Core Domain Set reflects the priorities of all relevant stakeholders, two groups of participants will be recruited: healthcare users and professional stakeholders (including clinicians, researchers, and commercial representatives). The method used to arrive at a Core Domain Set will be an e-Delphi survey, consisting of two online questionnaire rounds, and an online consensus meeting.

This new Core Domain Set would mean that all clinical trials for electrical stimulation-based interventions for tinnitus would use and report the same set of agreed outcomes. Standardised reporting would make comparison between studies easier, especially for meta-analysis and Grading of Recommendations Assessment, Development and Evaluations (GRADE) assessment as part of systematic reviews, improving the clarity on the knowledge produced and the interpretations of the merits of each intervention, leading to improvement in treatments for tinnitus and in turn management of tinnitus patients. In order to comply with GRADE, the final outcome set will be limited to a maximum of seven outcomes.

## Abbreviations

BRC: Biomedical Research Centre
CI: Chief Investigator overall
ENT: Ear, Nose and Throat
GCP: Good Clinical Practice
NIHR: National Institute for Health Research
NHS: National Health Service
PI: Principal Investigator at a local centre
PIC: Patient Identification Centre
PIS: Participant Information Sheet
REC: Research Ethics Committee
R&D: Research and Development department
tACS: Transcranial Alternating Current Stimulation
tDCS: Transcranial direct current stimulation
TMS: Transcranial magnetic stimulation
tRNS: Transcranial random noise stimulation
UoN: University of Nottingham
